# Improving the Sustainable Usage Intention of Mobile Payments: Extended Unified Theory of Acceptance and Use of Technology Model Combined With the Information System Success Model and Initial Trust Model

**DOI:** 10.3389/fpsyg.2021.634911

**Published:** 2022-01-10

**Authors:** Xin Lin, Kwanrat Suanpong, Athapol Ruangkanjanases, Yong-Taek Lim, Shih-Chih Chen

**Affiliations:** ^1^Northeast Electric Power University, Jilin City, China; ^2^Chulalongkorn Business School, Chulalongkorn University, Bangkok, Thailand; ^3^Chulalongkorn University, Bangkok, Thailand; ^4^Kunsan National University, Gunsan, South Korea; ^5^National Kaohsiung University of Science and Technology, Kaohsiung City, Taiwan

**Keywords:** information system success model (ISS), extending the unified theory of acceptance and use of technology (UTAUT2), sustainable usage intention, mobile payment, initial trust model

## Abstract

Under the background of global cross-border mobile commerce (m-commerce) integration, the importance of cross-border payment research is becoming increasingly prominent and urgent. The important value of this study is to empirically research the influence power of key elements in using two different mobile payment (m-payment) platforms in Korea. The extended unified theory of acceptance and use of technology (UTAUT2) has been widely applied in various studies because of its strong interpretive power. In Korea, there are a few empirical studies on Chinese users. Based on a survey of 908 Chinese participants (486 WeChat Pay’s Chinese users and 465 Kakao Pay’s Korean users) in Korea, this study is one application extending UTAUT2 by incorporating multi-group and multi-model constructs: UTAUT2, information system success (ISS) model, and an initial trust model (ITM), considering a multi-group analysis with some mediating variables (payment difference). By comparing the two different payment platforms’ characters, this manuscript provides a set of targeted measures to ensure Chinese WeChat Payment platform decision-makers create effective long-term strategic policies for cross-border m-payments in Korea, and eventually, benefit cross-border m-commerce and economic cooperation in Southeast Asia.

## Introduction

Under the background of the global mobile commerce (m-commerce) integration, mobile payments (m-payments) have become increasingly popular in Southeast Asia. Compared to traditional cash payment means such as credit cards, mobile digital payment systems can help consumers to complete various types of online payments through digital terminal devices without having to be restricted by time and location (Liébana-Cabanillas et al., 2018). As innovation in payment technology, m-payments are defined as “any payment method that uses a digital device to activate, authorize, and confirm the exchange of transaction values in exchange for products and services” (Kim et al., 2009). For example, the rapid expansion of m-payment transactions in China has been attributed to WeChat Pay, and the Korean m-payment market has benefited from “third-party m-payment platforms” such as Kakao Pay (Shao et al., 2019).

Mobile payment has brought about a historic technological revolution in the field of financial payments, with far-reaching social and economic impacts on the payment ecosystem in Southeast Asia and the world at large. With the high growth level of m-payment penetration in many economic entities, m-payment not only brings convenience to consumers but also increases the volume of business for many companies and improves the overall transaction payment level of the relevant economic entities (Fan et al., 2018).

Despite the numerous benefits described above, consumers are still limited by significant trust factors in accepting m-payment usage scenarios. For example, many traditional consumers continue to worry about the security and reliability of m-payment usage scenarios, because m-payment involves privacy information such as the user’s property account, credit card number, ID number, and account flow amount (Kim et al., 2010b). Previous relevant studies mainly analyzed the influence of various quality factors and initial trust (IT) on user behavior in m-payment scenarios (Liebana-Cabanillas et al., 2015). But, few studies empirically analyzed the antecedents of both various quality factors and IT.

Previous studies investigated the different factors affecting users’ adoption of m-payments, but there are still deficiencies to be filled. First, the research on m-payments is mainly concentrated in the financially developed countries, such as China and the United States (Fan et al., 2018), South Korea and the United States (Jung et al., 2015), China and South Korea (Lin et al., 2019), and India and the United States (Queiroz and Wamba, 2019), but these studies are still unsystematic and scattered. Second, globally, m-payment is a new technology. Relevant studies mainly focus on empirical analysis of consumers’ early willingness to adopt this technology, and few studies examine the stage after the use of new technology (Lin and Wu, 2021; Lin X. et al., 2021; Lin X. C. et al., 2021). Third, m-payment can be divided into two categories: short-range payment system (to pay for products or services by connecting electronic digital terminals to 4G network) and short-range payment system (to pay for products face to face in physical stores through payment technologies such as short-range communication and near-field communication (NFC), and short-range payment through mobile phones (Gerpott and Meinert, 2017). Previous relevant manuscripts either only studied the willingness to adopt m-payment with no difference between the two technologies (de Kerviler et al., 2016) or only studied the willingness to use a proximity payment system (Khalilzadeh et al., 2017; Verma et al., 2020).

Among the above-mentioned major e-commerce marketplaces, China’s e-commerce market was valued at USD 633.9 billion in 2018, with m-payments being one of the most popular payment modes. By 2023, total revenue is expected to grow by 11.6%, reaching total revenue of USD 10,945 billion. This means China’s e-commerce area is the fastest growing economic region and will remain in a leading position until 2023. Clearly, the trend of the transfer of e-commerce purchasing power from the European Free Trade Area, the United States Economic Area to Asia-Pacific Economic Cooperation (APEC) has begun. Due to the growth of APEC’s e-commerce purchasing power and the popularity of the Internet, especially gaining access to mobile devices, more consumers are utilizing m-payments (CNNIC, 2019).

The continuous growth of Korea’s m-payments is the main driving force for the continued growth of Korea’s, and even the world’s economy. Faced with Chinese m-payment consumers’ exponential growth and driven by the geographical advantages and high e-commerce penetration of APEC and China, Korean m-payment service providers are gradually realizing simply retaining existing Korean customers is not enough. To improve the Chinese users’ usage attitude effectively and rapidly toward Korean m-payment platforms, it is essential to concentrate on vital elements affecting the usage intention of Chinese consumers in using Korean m-payment systems.

Considering the above research gaps, this study developed the extended unified theory of acceptance and use of technology (UTAUT2) theoretical model integrating information system success (ISS) model and ITM for m-payment usage attitudes in China and Korea and tested the relationship between the constructs of the related models with a sample of 486 Chinese and 465 Koreans. This theoretical model not only studies the antecedents such as trust, quality, and payment conditions but also assumes the relationship between these constructs and their impact on use intention. The main purpose of this study is to fill the deficiencies in the following three aspects. First, this study focuses on several key antecedents to enhance the willingness to use third-party m-payment systems in China and South Korea. Second, this study examines the mediating effect between various antecedents and continuous intention of PE in the third-party m-payment platforms of China and South Korea. Finally, this study empirically analyzes the differences in the impact of cross-cultural comparison between the UTAUT2 integrated ISS model and ITM. The expected results of the manuscript can effectively fill the shortcomings of the existing literature and provide a more comprehensive theoretical and practical contribution to the development of the third-party m-payment system in China and South Korea.

The rest of this article is organized as follows: the second part comprehensively introduces the concept of m-payments and the existing research literature of the three information system models (ISMs) in the article; the third part expounds on the research theory and methods, research assumptions, puts forward the corresponding UTAUT2 integration model, and theorizes as to the potential relationship between facets; the fourth part introduces the research methods, data collection, analysis, and results, and discusses the results of statistical analysis; and the fifth part summarizes the research results, research contributions, practical impact, and suggestions for future research directions.

## Background and Literature Review

### Mobile Payment

Mobile payment refers to any online transaction explicitly initiated, granted, registered, and confirmed through mobile terminal equipment (Venkatesh et al., 2012; Dahlberg et al., 2015). Worldwide m-payment has stimulated a subversive revolution in the socio-economic field and has had a profound historical influence on the global payment system. The m-payment transactions are usually completed over long distances *via* network terminal electronic devices in the form of short message delivery, wireless billing, cellular networks, user-operated bills, and credit cards. As a result, m-payment systems can efficiently process m-economical transactions by various wireless technologies. The m-payment has a high penetration rate around the world, not only allowing convenient payment by consumers but also bringing economic returns to reduce transaction costs for companies that provide products and services and rapidly improving the overall national service levels for financial transactions (Phonthanukitithaworn et al., 2016).

According to the relevant literature, m-payment transactions contributed 4.6% toward global GDP in 2018, with a specific economic value of USD 3.9 trillion (CNNIC, 2019). Wang et al.’s (2017) research showed that cross-border m-payment between different countries played a significant role in promoting international trade integration. In particular, the development of cross-border m-payment platforms helped promote m-payment transactions and payments in APEC. In 2020, China’s cross-border transactions’ revenue is expected to be USD1.71 trillion, accounting for 37.6% of China’s foreign trade import and export volume. In 2023, global m-payment technology will grow to USD 4.8 trillion (4.8% of global GDP) (CNNIC, 2019). The integration of m-payment businesses even makes the sustainable development of the Southeast Asian cross-border consumer market in the process of rapid integration possible.

From what has been mentioned above, it is not difficult to make the following observations. First, in the context of the large-scale popularization of global m-payment platforms and rapid preemption of multinational markets, Korean m-payment platform service providers increasingly feel simply maintaining existing Korean customers is not enough. Second, a comprehensive analysis of the elements affecting the Korean users’ usage intention of Korean m-payment systems can increase the willingness of more consumers to use Korean m-payment platforms. Consequently, scholars have been trying to determine the elements influencing the willingness to use different m-payment systems. In the performance comparison process between Kakao Pay and Naver Pay, Lee and Kim (2017) revealed Kakao Pay showed limited applicability due to insufficient alliance merchants and also revealed a lack of reliability due to payment errors. The results showed Naver Pay also needed to improve reliability and greatly reduce errors in the early use and later payment process of the Naver Pay program. Due to the need for a tedious payment operation and a large amount of consumer personal information, it is easy to make users feel weary. By analyzing the response of the international cross-border consumer market of Kakao Pay and Samsung Pay, Son and Kim (2018) discovered management strategies can ensure the sustainable development of rapid technology. By comparing the conversion intention between Samsung pay and Kakao Pay, Lee et al. (2017) firmly believed the more benefits obtained after the conversion of different m-payment platforms, the stronger the convenience experience brought by the conversion of different m-payment platforms, the safer the conversion process, the stronger the conversion intention of consumers between different m-payment tools will be.

Additionally, similar to Kakao Talk, WeChat Pay binds the WeChat account to the user’s bank card and uses NFC or QR for payment services. This can be easily viewed online at any time through the APP *via* WeChat’s official account. Other previous studies indicated some scholars used various representative structural equation models as the basic structure of the research, including technology acceptance model (TAM2), UTAUT, and UTAUT2, and any element that might influence the willingness to use m-payments (Alalwan et al., 2018; Liébana-Cabanillas et al., 2018). This study’s conclusion further confirms the research significance of this manuscript. In other words, it is necessary for this manuscript to integrate the models and analyze the Chinese and Korean m-payment platforms and to better promote the win-win cooperation of Chinese and Korean m-payments.

### Extended Unified Theory of Acceptance and Use of Technology Model

How to classify and explain the influencing elements affecting the consumers’ voluntary usage intention of new m-payment platforms has become the most important study area of information technology systems (Swanson, 1988). Davis (1989) and Venkatesh and Davis (1996) successively put forward some theoretical structures of information technology, for example, the TAM and UTAUT model, to explain the elements directly affecting the willingness of a new m-payment platform. Venkatesh et al. (2012) expanded UTAUT from a perspective of new technology perception of users by absorbing price value (PV), hedonic motivation, and habit and finally proposed UTAUT2 that further improved the interpretation ability of UTAUT. Correspondingly, other researchers (Martins et al., 2014) also suggested in future research, a more complete UTAUT2 model must be used for further analyzing the relevant factors affecting the consumer usage intention of m-payment.

The UTAUT2 model has been applied to analyze and test influencing elements of m-commerce (Chopdar et al., 2018), m-transactions (Farah et al., 2018), and m-banking (Khan et al., 2017) usage intention. Studies also showed UTAUT2 was an effective model for understanding the usage intention of m-payment (Wu and Lee, 2017). The existing research focused on the integration of various theoretical structures to study the usage willingness of new information technology. Particularly, numerous studies on the application of m-banking integrated UTAUT2 with other theories (Lin et al., 2019), showing the necessity and importance of using other theories to make up for the theoretical gaps in information technology.

Although the majority of the literature mostly used age, gender, and experience as moderating variables, few academics attempted to improve the model with other structures to improve its accuracy in the m-payment area (Baptista and Oliveira, 2015). Therefore, recent research analysis (Oliveira et al., 2014) suggested integrating distinctive models to obtain a more complete view to accomplish their research goals. This research will use UTAUT2 in combination with other important information technology models as a theoretical framework to evaluate the elements influencing consumer acceptance of the m-payment platform in Korea. Consequently, the next study should research integrating various information technology models into UTAUT2 and then analyze which factors can influence the m-payment platform users’ usage willingness.

### DeLone and McLean’s Information System Success Model

Information system success explains how system and information qualities affect users’ usage willingness and user satisfaction (US), leading to the influence of individual willingness (DeLone and McLean, 1992). Moreover, DeLone and McLean (2003) improved an upgraded model that incorporated quality of service. From then on, the upgraded ISS was extensively applied to assess the usage willingness of dissimilar m-payment systems. In our article, ISS is applied to confirm some promoters of usage willingness in the m-payment system. Tam and Oliveira (2017b) proposed ISS moderated by the cross-cultural dimension, revealing the relationship between personal performance and m-banking is mediated by diverse-cultural effects on the usage of m-banking. Hence, mobile bank managers were provided new insights from the mediating influence of cultural effects, which is very important to recollect former users and further attract potential users by applying strategies.

Mobile payment is an advanced information payment technology, which has rarely been studied by researchers, especially the different groups of WeChat Pay’s and Kakao Pay’s customers in Korea. Accordingly, ISS should be utilized and generalized as a theoretical model of m-payment with other information models.

### Initial Trust Model

Initial trust emphasizes the “usage intention of the customer to take advantage of trust in satisfying a demand without pre-experience, or reliable, profound information” (Kim and Prabhakar, 2004). Accessibility, adaptability, and potential profits (the function of service utility) can be attributed to the foundation of IT (Koufaris and Hampton-Sosa, 2004). Therefore, Kim et al. (2009) found a model using ITM, whereby the IT of the mobile bank could be explained by a structural guarantee, trust tendency, and corporate reputation.

Some scholars (Kim, 2012; Zhou, 2014) contended IT has been proven to be an important factor influencing the first adaptation decision of consumers because the stable usage intention can be formed only after the IT is established. Three categories of the element are classified as follows: the first element is linked with the features of m-payment. The usage intention of consumers will depend on IT to a certain extent. Of course, structural factors are effective in influencing IT (Lin, 2011). The second element is closely related to the reputation of the company. Corporate reputation is also an important factor affecting IT because it reduces the risk of potential price information asymmetry and forms an after-sales guarantee after completing the m-payment transaction process (Li et al., 2008). The third element is combined with users’ trust tendency. Personal trust tendency reveals a psychological trend of users, which also has an important impact on IT.

The initial trust model was used in various researches to judge or predict the usage intention of the m-payment system, for instance, m-shopping, m-banking, m-commerce, and m-payments (Shankar and Jebarajakirthy, 2019).

### Research Model

As mentioned above, originally suggested by DeLone and McLean (1992), system quality is interpreted as the quality expressed in the complete function of the system, therefore it can be perceived by individuals (DeLone and McLean, 2003). Furthermore, Venkatesh et al. (2012) interpreted performance expectation (also performance expectancy, PE) as “the extent to which technology will assist customers when completing certain tasks.” Obviously, powerful navigation, a clear outline, and a responsive interface may be crucial for adopting m-payments. Thus, system quality directly affects PEs.

Bhattacherjee (2002) pointed out if consumers can experience better system performance, it will significantly increase US and thus generate continuous use intent. Notably, the better the system quality, the greater it can markedly improve US, and the more it can make up for the limitations of mobile device size. Pyo and Kim (2014) found Kakao Talk users are more interested in ease of use and high-speed accessibility, and system quality significantly impacts satisfaction. Sharma (2019) pointed out users may not trust the m-payment platform’s ability to provide high-quality system services, which may make it harder to use the device, according to a group of users who are not able to meet the user’s expectations. The following hypotheses are given:

H1a: System quality significantly influences user satisfaction.
H1b: System quality significantly influences performance expectancy.

Information quality (IQ) includes comprehensibility, accessibility, sufficiency, accuracy, feedback report, and other characteristics (Sharma, 2019). Clearly, information quality is a vital element to determine the usage intention toward information mobile technology. DeLone and McLean (2003) pointed out that information quality was also a crucial part of the m-payment information platform, which was the most basic communication ability of Internet buyers and sellers. Interpreted as the inherent quality of the information itself, such as accuracy, reliability, and completeness, information quality significantly influences PE (Tam and Oliveira, 2016). The m-payment consumers always want to gain the whole transaction records, accurately, and timely. After all, users will also be concerned with the m-payment transaction is complete. If there is no receipt, the payer cannot obtain proof of the payment transaction, so it is difficult to ask for a refund when the goods are not ideal. The m-payment consumers generally believe a lack of transaction-related information is risky. They are not sure whether the payment has occurred or not, and they are not sure about the payment (Mallat, 2007). This extra difficulty of tracking past payments may additionally make consumers feel service suppliers do not provide sufficient functional investment in m-payments. The m-payment consumers’ PE of consumers will be influenced.

Information quality, on the other hand, may also affect customer satisfaction. Existing studies reported how IQ affected US, m-banking, and the virtual community (Elliot et al., 2013). The following hypotheses are given:

H2a: Information quality significantly influences user satisfaction.
H2b: Information quality significantly influences performance expectancy.

Service quality (SQ) refers to some features of service aids (such as responsiveness, credibility, simplicity, and technical ability, etc.) obtained by consumers from the information transfer department and technical support department (DeLone and McLean, 2003). In addition, service quality will also affect the US wireless business transactions (Gounaris et al., 2010), virtual travel societies, and mobile instant messaging (Elliot et al., 2013).

Clearly, information systems cannot be fully evaluated effectively without reference to the quality of service. All quality factors, including SQ, will help users to evaluate their PE correctly. The following hypotheses are given:

H3a: Service quality significantly influences user satisfaction.
H3b: Service quality significantly influences performance expectancy.

DeLone and McLean (2003) defined US as “the degree to which an application platform can create value for internal or external consumers.” This means US reflects consumers’ subjective feelings accumulated in full cooperation with mobile suppliers (San-Martín et al., 2013). Furthermore, prior research proposed satisfaction is a decisive determinant of the willingness to use continuously (Zhou, 2014; Lee et al., 2015).

The US of the ISS model reveals the positive correlation between PE and usage willingness (Au et al., 2008). According to previous literature, PE is positively correlated with the relevant models (DeLone and McLean, 1992). UTAUT2 also explained that the influence of enhancing US to user’s usage willingness was clearly significant, and US was also affected by PE. Therefore, the increased PE will positively increase US and ultimately influence the acceptance intention.

On this basis, Tam and Oliveira (2016) revealed an affirmative correlation between US and m-banking service intention is established by integrating UTAUT2 into ISS. They also confirmed service quality directly affects the performance, and US by confirming satisfaction is the consumers’ feeling from the total qualities provided by m-payment provider qualities in the wireless economic commerce environment. The following hypotheses are proposed:

H4a: Performance expectancy significantly influences user satisfaction.
H4b: User satisfaction significantly influences usage intention.

Structural assurance (SA) refers to the trust structure framework based on institutions, which is determined by laws, credit guarantees, and the industry regulations existing in a certain environment (McKnight et al., 1998). Judged by the above views, we conclude that IT comes from people’s sense of security in the process of online banking transactions under the dual effects of relevant social institutions, industry laws, government supervision, contract and offer, and the online structure of online banking. If the information of the counterparties is incomplete, the role of these structural security measures is essential to consumers’ IT.

In the m-payment transaction environment, a structural guarantee ensures the reliability of financial transactions, the protection of personal privacy, and transaction confidentiality. With promises, deals, rules, contracts, laws, managed services, and other forms of structural guarantees, the IT in a transactional relationship can be enhanced (McKnight et al., 2004). It can improve the user’s IT, considering that the user wants to be guaranteed and avoid the risks and uncertainties affecting information, finance, etc. (McKnight et al., 2002).

Of course, the structural guarantee has been proven to affect the trust of the bank (Moin et al., 2015), electric commerce (Alqatan et al., 2016), and m-banking (Yu and Asgarkhani, 2015), which regards manuscript reports as structural guarantees. The following hypothesis is suggested:

H5a: Structural assurance significantly influences initial trust.

Personal propensity to trust (PPT) refers to users’ natural tendency to trust new technology. Consumers with a natural trust tendency have a greater tendency to trust mobile banks (McKnight et al., 2002). Personal trust tendency is an attribute characteristic and experience formed by one’s cultural background and psychology (Lee and Turban, 2001). When people make judgments about services without prior knowledge, those who are more inclined to trust may think services are reliable. Many studies on the IT of online banking transactions show the individual’s trust tendency may positively influence the establishment of trust in m-banking (Gefen, 2000).

Therefore, personal trust often does not have the experience of dealing with the trustee and relies on trust expectations. In the IT situation, personal trust significantly impacts IT. Personal trust tendency is a kind of trust formed from small to large. This kind of personal trust is generally considered to be direct and dependent behavior (Kim and Prabhakar, 2004). Research by Wu and Lee (2017) reveals the personal trust tendency of customers will positively influence adoption willingness when a company offers reliable and accurate services. The following hypothesis is stated as follows:

H5b: Personal propensity to trust significantly influences initial trust.

Firm reputation (FR) refers to a company’s ability to provide an effective service to its customers and the reliability of customers’ participation in the company’s business (McKnight et al., 1998). The company’s reputation improves consumers’ trust in its new services and helps to comprehensively improve the trust of potential consumers in new service transactions (Kim et al., 2009). Therefore, enterprise reputation is one important element of IT. It directly influences users’ willingness to adopt related services.

The existing research shows that the most important thing is customers initially trust the reputation of the enterprise, rather than taking trust behavior according to the actual scale of the enterprise (Wu and Lee, 2017). Therefore, many well-known enterprises actively provide after-sales support for customers, timely publicize, and improve the high-tech image of the enterprise, and persuade consumers to believe that the well-known enterprise has sufficient technical strength and core competitive advantages, thus greatly improving the consumer trust of the enterprise’s mobile operation platform. The following hypothesis is submitted:

H5c: Firm reputation significantly influences initial trust.

Initial trust points out the user’s intention to bear unexpected losses to meet the demand, without the use of experience or reliable and referential information (McKnight et al., 1998; Kim and Prabhakar, 2004). IT guarantee consumers eventually reach the desired outcome (Pavlou et al., 2003). Especially in the m-payment environment, the trust of consumers strengthens the individual’s expectation of the product’s usefulness or performance (Bock et al., 2012). Prior research also indicated that trust would promote volunteering that influences the perceived usefulness of the web platform (Saura et al., 2020). If the service provider is not trusted to provide dependable m-payment services, the positive adopters are more likely to suffer losses after adopting m-payments because the service supplier is speculative. Therefore, the IT factor may positively affect the PE of consumers (Dishaw and Strong, 1999). The following hypothesis is submitted:

H6a: Initial trust significantly influences performance expectancy.

Kim et al. (2010a) studied the impact of IT on m-payment usage acceptance. They confirmed the elements of IT, together with the relative benefits of m-payments, structural guarantees, corporate reputation, and users’ propensity to trust. Hung et al. (2012) showed that trust played a critical role in mobile shopping’s persistent intention. Due to the mobile networks’ vulnerability, the mistrust of mobile providers, and m-payment systems, m-commerce has greater doubt and hazards. Viruses and Trojans can also infect mobile platforms. Under the background of m-payments, a purchase is influenced by safety and trust issues, so more risk and more mistrust should be considered (Chen, 2013). M-payment consumers have reason to worry whether the m-payment platform can safely transfer and store their own credit card accounts, passwords, location privacy, and other privacy information (Mamonov and Benbunan-Fich, 2015). Therefore, we believe that IT may influence the sustainability of m-transactions and thus make the hypothesis as follows:

H6b: Initial trust significantly influences usage intention.

Performance expectancy refers to the degree to which a user considers adopting an m-platform contributes to his work performance (Venkatesh et al., 2003). In previous literature, if individuals figure out that the profit of using new technology outweighs the disadvantages, they will be more inclined to accept and continue to adopt the technology (Venkatesh et al., 2012). Unambiguously, in a large number of m-payment scenarios, PEs are found to directly affect the user’s usage intention of the relevant information system (Baptista and Oliveira, 2015).

In the m-commerce environment, consumers will judge the effectiveness of using the m-payment application platform to help complete their business transactions. Clearly, PE is one critical element in the process of consumer evaluation. Many previous studies (Faqih and Jaradat, 2015) explicitly support the positive influence of the willingness to use m-commerce. In addition, more research results show that PE plays a critical role in affecting the willingness to use m-payment platforms (Morosan and DeFranco, 2016). The hypothesis is:

H7: Performance expectancy significantly influences usage intention.

Effort expectancy’s (EE) definition (Venkatesh et al., 2003) is “the degree of ease associated with using the system.” In a large number of studies involving UTAUT2, the expected workload has been generally considered a vital precondition for the expected work (Venkatesh et al., 2003, 2012; Slade et al., 2015). That is, the influencing factors of consumers’ willingness to accept a new platform are not only the benefits of the platform itself, but also the difficulty and effort of using the system. The ease of access to a system tends to stimulate users to adopt it (Oliveira et al., 2014; Dwivedi et al., 2019). Under the background of m-payments, the EE will be regarded as the capability to carry out a certain mobile business function with the least amount of work. Reasonable work expectations can make consumers feel very comfortable when they carry out the m-commerce transaction.

In addition, the particularity of m-banking also forces system operators to have some basic finance knowledge and related operational skills. Therefore, efforts are expected to effectively influence determining customers’ willingness to use an m-payment platform system (Alalwan et al., 2016). Many m-banking studies demonstrated the factors captured by effort expectations positively affect measuring customers’ usage intention of m-banking (Gu et al., 2009). The interaction interface, function design, and computing power of m-banking can directly influence the consumers’ willingness to adopt. The interaction interface, function design, convenient navigation, and the computing power of m-banking can directly affect the user’s willingness to adopt (Venkatesh et al., 2003; Kim et al., 2009). Therefore, the following hypothesis is given:

H8: Effort expectancy significantly influences usage intention.

Social influence (SI) indicates some extension to which platform users’ important social relations (e.g., family, friends, or leaders) have faith in the new m-payment system should be adopted (Venkatesh et al., 2012; Tam and Oliveira, 2016). Social impact reveals the impact of individuals on the adaptation of technology by their social relatives. Users often consider the opinions of others when choosing a new technology. Supposing the attitude of his social relatives is active, users will accept it; on the contrary, negative attitudes will affect users’ decision not to adopt.

The social preferences and values coming from family relatives, friends, and neighbors often profoundly change users’ views and opinions (Rana et al., 2017). Especially when the present user intends to change from using one technical service to another technical service, the user’s willingness to change will be easily influenced by peers and influence of family members (Baptista and Oliveira, 2015; Dwivedi et al., 2019). Under the background of mass media dominating the online world (Kapoor et al., 2018), the impact of social relations may not only continue the usage intention of old technology but also guide users to new technologies recognized by social relations (Williams et al., 2015). Through multicultural surveys between Australia and Thailand, Mortimer et al. (2015) found that even in different cultures, social impact can become a significant element in the usage intention of a new platform. Moreover, under the background of Saudi Arabia’s electrical commerce, prior research also proves the active influence of SI on the adoption willingness of m-banking (Al-Husein and Asad Sadi, 2015). The hypothesis is given:

H9: Social influence significantly influences usage intention.

Facilitation (FC) reflects a positive and significant impact of the infrastructure related to organization and technology on the use of online banking, such as consumer expertise, related operational skills, and platform resources (Venkatesh et al., 2003). In fact, the enhancement of the willingness to use m-payments requires online banking to train users to have specific operation skills, provide service resources and basic hardware and software conditions of high matching financial systems (Alalwan et al., 2015). The necessary knowledge reserve and skill accumulation play many roles in adopting m-banking services, thus affecting the usage intention. Previous literature studies pointed out convenience significantly impacts usage willingness (Alalwan et al., 2016). The hypothesis is:

H10: Facilitating conditions significantly influence usage intention.

Hedonic motivation’s (HM) definition mentions a degree of enjoyment in the process of adopting m-banking, which is a vital pretest factor affecting consumer willingness to use a new technology (Van der Heijden, 2004); hedonic behavior is essentially a non-functional and personality emotional variable, which is completely based on consumers’ emotional cognition (Malik et al., 2013). In other words, the pleasure gained from using new technologies significantly promotes usage intention (Alalwan et al., 2015). The higher the degree of entertainment the mobile platform brought, the greater willingness customers will have (Zhang et al., 2012).

In addition, many previous studies revealed hedonic motivation was positive in predicting usage intention in various m-payment technology application scenarios (Morosan and DeFranco, 2016; Alalwan et al., 2017). When consumers find the existing m-payment technologies bring enough effective comfort, satisfaction, and pleasure, they often do not switch their use intention to other competitive payment platforms (Karjaluoto et al., 2010). Therefore, many research conclusions have revealed the satisfaction or pleasure obtained by consumers from using a specific m-payment platform is an extremely vital element of the willingness to use the technology (Morosan and DeFranco, 2016; Alalwan et al., 2017; Dwivedi et al., 2019). Accordingly, the hypothesis is submitted:

H11: Hedonic motivation significantly influences usage intention.

The price value is a cognitive balance comparing the profits from m-banking platforms and the financial price of adopting m-banking services that consumers experience (Venkatesh et al., 2012), including m-payment service operator costs, equipment costs, after-sales costs, purchase and sale costs, and other factors. PV is optimistic because the profits of adopting m-banking outweigh the associated currency costs.

Since the profits of adopting m-bank are larger than related economic value, the PV is positively correlated. As Alalwan et al. (2017) indicated, the higher the PV level, the more motivated customers are to continue to adopt a certain technology. Further, after UTAUT2 introduced PV, prior research pointed out that there was an important positive correlation between PV and behavioral intention (Alalwan et al., 2017). Therefore, considering the potential profits of adopting the electrical commerce apps, consumers may reassess whether the relevant transaction costs are reasonable. If the potential revenue is significantly greater than the foreseeable cost of adopting m-payment applications, consumers are more inclined to adopt m-commerce solutions. Otherwise, users who cannot afford to continue using the upgraded technology will not express interest in continuing to adopt it. Given the above situation, this hypothesis can be given:

H12: Price value significantly influences usage intention.

Habit (HA) reveals various outcomes of past practice, and the frequency of previous acceptance behavior is reflected to be one main element of current behavior (Ajzen, 2002). The empirical research of Eriksson et al. (2008) reveals that a positive correlation between online banking and customer habits is an important factor for US users to accept online m-commerce. Pavlou and Fygenson (2006) contended habits could be the principal determinants of prior behavior.

Consumers are less likely to change acquired habits and may resist any new and unfamiliar interactions with m-banks (Chemingui, 2013); this research further leads consumers to hesitate to adopt new apps or platforms, for instance m-payments (Antón et al., 2013). Actually, as an unconscious factor, previous experience, and habits largely hinder consumers’ intention to use a new platform, because they tend to depend on past experience rather than cognitive reasoning when making decisions (Zhang et al., 2017). In this research, following the structure of UTAUT2, habits have taken a positive role in motivating online banks to take actions. Therefore, the hypothesis postulates:

H13: Habit significantly influences usage intention.

Khan et al. (2017) finally determined the moderating effect of cultural factors by revealing a certain relationship between UTAUT2 variables. Through an adjustment analysis and segmentation test, Zhang et al. (2012) argues the use intention model of m-payment and divides it into two cultures to determine the regulatory role of different ethnic PVs in m-commerce. The results reveal UTATU2 is in good agreement with the data. Lee et al. (2017) found in the UTAUT model, the role of government has an adjusting effect on the determinants of adoption willingness. According to the above results, we examined the moderating effect of different ethnic consumers in an integrated UTAUT2 model. We assume there are some dissimilarities, which adjust the influence of these determinants on users’ intention to use. In the empirical investigation of this manuscript, it is necessary to compare and analyze WeChat payment in China and Kakaopay, the largest m-payment platform in South Korea to study the subjective and objective factors affecting the sustainable development of Kakao Pay as comprehensively as possible. In the near future, Kakao Pay and WeChat payment will provide remote payment services, respectively, to support the m-commerce payment of up to 450 million Chinese and Korean consumers. Facing the growing Chinese consumer group, the successful survival of the Korean m-payment system is mainly affected by important core factors, such as US, PE, compatibility between technical characteristics and task requirements, etc. The cultural differences between China and Korea will certainly affect the usage intention of m-payment services by different consumers to some extent. In the process of globalization of m-payment technology, the importance of cross-cultural research is obvious. Thus, we test the hypotheses:

H14: The impact of initial trust on usage intention differs between WeChat Pay and Kakao Pay Chinese consumers.

H15: The impact of user satisfaction on usage intention differs between WeChat Pay and Kakao Pay Chinese consumers.

H16: The influence of performance expectancy on usage intention differs between WeChat Pay and Kakao Pay Chinese consumers.

H17: The influence of effort expectancy on usage intention differs between WeChat Pay and Kakao Pay Chinese consumers.

H18: The effect of social influence on usage intention differs between WeChat Pay and Kakao Pay Chinese consumers.

H19: The impact of facilitating conditions on usage intention differs between WeChat Pay and Kakao Pay Chinese consumers.

H20: The impact of hedonic motivation on usage intention differs between WeChat Pay and Kakao Pay Chinese consumers.

H21: The impact of price value on usage intention differs between WeChat Pay and Kakao Pay Chinese consumers.

H22: The impact of habit on usage intention differs between WeChat Pay and Kakao Pay Chinese consumers.

**FIGURE 1:**
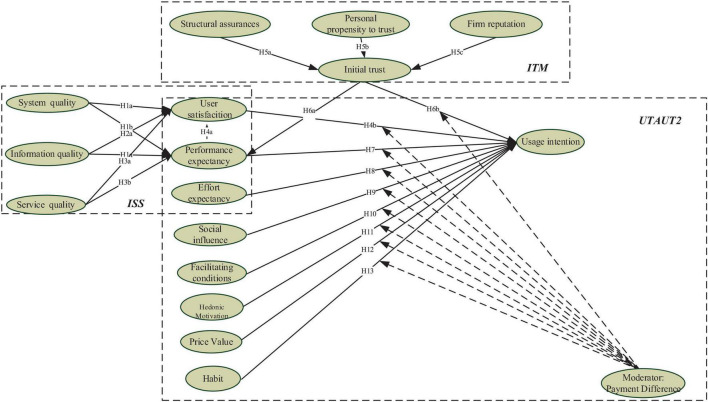
Research model.

According to the above hypotheses development, we proposed the research model for this study (as shown in Figure 1).

## Data Collection and Results

The constructs in this study are measured using 7-point Likert scales drawn and revised from existing studies (e.g., DeLone and McLean, 2003; Venkatesh et al., 2003, 2012; Koufaris and Hampton-Sosa, 2004). We employed the back-translation procedure suggested by Brislin (1970), using focus groups to ensure a match between the original wordings and their translation. Subsequently the measurement items were translated into English and double-checked for veracity of meaning from Chinese to English by two native English speakers.

From the beginning of July to the end of August 2019, through face-to-face interviews with experienced users coming from Chinese students in Seoul University and local Kakao Pay’s Korean experienced users, the survey was conducted for 8 weeks.

We used the two-process method (Anderson and Gerbing, 1988) to evaluate the collected data. In the first step, convergence and discriminant validity are examined. Second, the measurement model between the integrated structures is evaluated. To test the fitting of the measured values and structure modeling, the integration structure was tested using all of the WeChat Pay and Kakao Pay’s Korean consumer data. Hooper et al. (2008) offered model fitting cooperation as a fitting index and proposed the following indexes as fitting indexes.

As the m-payment industry of the Seoul cultural business circle and many universities is far more mature than other regions in South Korea, we quickly obtained ideal samples. Meanwhile, to ensure the measurement followed the direct behavioral practice of the object, some participants not using WeChat Pay and Kakao Pay were strictly excluded. Moreover, by modifying the ambiguous part of the questionnaire, this research guarantees each respondent can fully comprehend every question of the questionnaire.

A survey of 1,200 questionnaires was issued and 1,143 copies were collected (response rate 86.46%). After 192 responses were discarded, 951 samples (85.33%) were eventually used for deterministic analysis (486 WeChat Pay’s Chinese consumers 465 and Kakao Pay’s Korean consumers) because of missing key data or lack of corresponding m-payment experience. The final data is sufficient to define the difference in the middle of the two object groups, although the sample sizes are not identical.

Every item was asked on a five-point Likert’s scale (Table 1). For instance, “strongly reject” to 5, “strongly agree.” In the subsequent empirical analysis, Cronbach’s α was applied to calculate the reliability of the measurement method using IBM SPSS 24.0 (IBM Corp., Armonk, NY, United States), and construct validity was evaluated by examining the factor structure and intrinsic relevance of each construct. To test the research hypotheses, we used IBM Amos 24.0 (IBM Corp., Armonk, NY, United States) and determined the causal relationship between core variables through a significance value and standard coefficient. For testing each hypothesis, the entire sample is used to analyze the integration model before the hypothesis verification test.

**TABLE 1:** Sample characteristics (entire samples).

Characteristics		Frequency	Percentage (%)	WeChat Pay’s		Kakao Pay’s	
Gender	Male	433	45.53	219	45.06%	214	46.02%
	Female	518	54.47	267	54.93%	251	53.98%
Age	Below 20	93	9.80	55	11.30%	38	8.20%
	20–30	391	41.11	183	37.65%	208	44.73%
	30 –40	351	36.90	182	37.45%	169	36.34%
	40 –50	65	6.80	35	7.20%	30	6.50%
	Over 50	51	5.40	31	6.40%	20	4.30%
Education	High school student/resident	91	9.60	51	10.50%	40	8.60%
	College student/student	417	43.80	207	42.60%	210	45.20%
	Graduate school or higher	443	46.60	228	46.90%	215	46.20%
Experience	Yes	951	100.00	486	100.00%	465	100.00%

### Reliability, Validity, and Measurement Model

Three steps are required to evaluate the convergence effectiveness of a measurement object against its related structures. First, the reliability of each index is evaluated by means of standardized load. Second, as a measuring item, Cronbach’s α and CR are used to measure the reliability of composites. Third, extracted average variance (AVE) measures the variance of variables caused by measurement error relative to the variance.

According to Table 2, Cronbach’s α and CR are above 0.70 (Nunnally, 1994), indicating the optimal validity measures explain the structure and higher level of comprehensive consistency. Moreover, convergent validity was measured by three dimensions of indicators: the standardized loadings signifying the relationship between some underlying elements and every indicator was statistically above 0.7, the Cronbach’s α values were significant at greater than 0.7 for the reliability of the integrated construct (Nunnally, 1994; Hair et al., 1998); each AVE value was greater than 0.5 (Fornell and Larcker, 1981).

**TABLE 2:** Convergent validity and reliability (entire samples).

Construct	Indicators	Standardized loading	Cronbach’s α	Composite reliability	AVE
SYQ	SYQ 1-4	0.768–0.888	0.889	0.890	0.669
IQ	IQ 1-4	0.821–0.858	0.900	0.901	0.694
SEQ	SEQ 1-4	0.799–0.866	0.903	0.904	0.702
US	US 1-4	0.805–0.861	0.899	0.900	0.691
PE	PE 1-4	0.777–0.865	0.894	0.896	0.683
EE	EE 1-4	0.810–0.871	0.905	0.906	0.706
SI	SI 1-4	0.772–0.873	0.901	0.902	0.699
FC	FC 1-4	0.802–0.862	0.898	0.899	0.690
HM	HM 1-4	0.808–0.848	0.894	0.896	0.683
PV	PV 1-4	0.809–0.853	0.897	0.897	0.686
HA	HA 1-4	0.810–0.864	0.896	0.897	0.686
SA	SA 1-4	0.816–0.862	0.906	0.907	0.710
PPT	PPT 1-4	0.807–0.868	0.903	0.904	0.701
FR	FR 1-4	0.818–0.836	0.900	0.901	0.696
IT	IT 1-4	0.844–0.890	0.918	0.926	0.757
UI	UI 1-4	0.760–0.860	0.889	0.889	0.667

In Table 3, discriminant validity indicates the difference between one principle and its related indicators and the second principle and its related indicators (Bagozzi et al., 1991). Fornell and Larcker (1981) found discriminant validity must be tested by evaluating the square root of each variables’ AVE in each construct and their correlation coefficients among other models.

**TABLE 3:** Discriminant validity (entire sample).

	SYQ	IQ	SEQ	US	PE	EE	SI	FC	HM	PV	HA	SA	PPT	FR	IT	UI
SYQ	0.82															
IQ	0.29	0.83														
SEQ	0.27	0.37	0.84													
US	0.54	0.62	0.36	0.83												
PE	0.56	0.55	0.32	0.69	0.83											
EE	0.11	0.19	0.14	0.19	0.11	0.84										
SI	0.14	0.19	0.12	0.20	0.14	0.44	0.84									
FC	0.16	0.20	0.17	0.25	0.16	0.46	0.42	0.83								
HM	0.16	0.16	0.11	0.19	0.13	0.41	0.41	0.49	0.83							
PV	0.08	0.18	0.10	0.19	0.15	0.39	0.40	0.48	0.38	0.83						
HA	0.11	0.24	0.16	0.22	0.17	0.44	0.42	0.50	0.40	0.45	0.83					
SA	0.22	0.28	0.22	0.32	0.37	0.22	0.24	0.27	0.20	0.22	0.30	0.84				
PPT	0.16	0.17	0.16	0.22	0.30	0.22	0.20	0.24	0.20	0.20	0.24	0.51	0.84			
FR	0.21	0.23	0.18	0.26	0.34	0.21	0.24	0.26	0.23	0.23	0.29	0.53	0.50	0.83		
IT	0.26	0.26	0.22	0.36	0.55	0.23	0.23	0.28	0.25	0.25	0.30	0.70	0.67	0.68	0.87	
UI	0.22	0.28	0.14	0.44	0.45	0.38	0.45	0.48	0.44	0.49	0.47	0.28	0.25	0.25	0.44	0.82

Table 3 shows for each data compared with the correlation between one structure and another, the variance between structures and each AVE’s square root is larger than any related correlation coefficient, pointing to good discriminant validity of each criterion (Fornell and Larcker, 1981). The correlation between constructs was exceeded by the diagonal values, proving the satisfactory construct validity of our measurement tool.

The IBM Amos 24.0 program (IBM Corp., Armonk, NY, United States) was utilized to evaluate the measurement and structural models of this research. The χ2/d.f.s are 1.299 and 1.408, GFIs are 0.927 and 0.919, AGFIs are 0.917 and 0.910, NFIs are 0.949 and 0.943, CFIs are 0.988 and 0.983, IFIs are 0.988 and 0.983, RFIs are 0.944 and 0.939, PGFIs are 0.816 and 0.824, PCFIs are 0.898 and 0.910, PNFIs are 0.862 and 0.873, RMRs are 0.050 and 0.061, and RMSEAs are 0.018 and 0.021. The results from the measurement and structural models support this association for each model. Twenty research hypotheses presented in this manuscript were tested by scanning electron microscope (SEM). For the parsimonious fitting index, the acceptable fitness minimum is exceeded here, which is a standard value. All the fitting indexes show the fitting results of the analyzed samples and the integrated model are satisfactory.

### Hypothesis Verification

After determining the measurement suitability and organization of the combined model, the structure was analyzed with Chinese samples and the Chinese path coefficient was evaluated as shown in Table 4. Judging by the *p*-value, 4 paths of the 20 paths (H3a, H3b, H6b, and H8; *p-*value of > 0.05) were rejected, and the other 16 paths proved statistically positive.

**TABLE 4:** Results of hypotheses tests (WeChat Pay’s Chinese consumer’s sample in Korea).

Hypothesis	Route	*T*-Value	Path coefficients
H1a	SYQ → US	5.237	0.260***
H1b	SYQ → PE	10.498	0.482***
H2a	IQ → US	6.525	0.324***
H2b	IQ → PE	10.269	0.459***
H3a	SEQ → US	0.641	0.025
H3b	SEQ → PE	0.213	0.008
H4a	PE → US	7.006	0.415***
H4b	US → UI	5.589	0.351***
H5a	SA → IT	7.597	0.347***
H5b	PPT → IT	7.719	0.360***
H5c	FR → IT	7.410	0.338***
H6a	IT → PE	10.417	0.438***
H6b	IT → UI	1.350	0.057
H7	PE → UI	4.162	0.291***
H8	EE → UI	0.535	0.020
H9	SI → UI	5.168	0.189***
H10	FC → UI	2.645	0.107**
H11	HM → UI	7.023	0.269***
H12	PV → UI	2.148	0.079*
H13	HA → UI	7.082	0.285***

*p-value < 0.05; **p-value < 0.01; and ***p-value < 0.001.

Chinese consumers’ usage intention revealed by ITM (β = 0.057), US (β = 0.351), PE (β = 0.291), EE (β = 0.020), SI (β = 0.189), FC (β = 0.107), HMM (β = 0.269), PV (β = 0.079), and HA (β = 0.285) explain 75.1% of the variance in adoption willingness. The influence on Chinese users shows the antecedent variables of the ITM, ISS model, and UTAUT2 model account for 61.7, 62.6, and 68.1% of the variance, respectively, which are related to the 75.1% explanatory ability of the comprehensive structure on use willingness. The measurement and structural model results are given, and a comprehensive model analysis is carried out with Kakao pay’s experience consumers sample as an example. Kakao Pay path coefficient between the basic hypotheses of the comprehensive model was properly evaluated. Judging by their respective *p*-values, 5 paths of these 15 paths (H3a, H3b, H4b, H7, and H8; *p*-value > 0.05) were unqualified, and the rest of the paths are statistically positive.

Kakao Pay consumers’ usage intention predicted ITM (β = 0.509), US (β = 0.061), PE (β = 0.050), EE (β = 0.040), SI (β = 0.213), FC (β = 0.278), HMM (β = 0.099), PV (β = 0.330), and HA (β = 0.097) explain Kakao Pay’s Korean consumers’ usage willingness for 76.7% of the explained variance. The influence of Kakao Pay’s Korean consumers shows the prerequisites of ITM, ISS, and UTAUT2 can explain the variance of 60.0, 64.1, and 66.8%, respectively, and the explanatory power of these three variables for the comprehensive model is 76.7%.

The analysis outcomes of the whole dataset are shown in Table 5. There are four paths (H3a, H3b, H6b, and H8), *p*-value > 0.05) not supported, and the other paths are significantly below the 0.05 level. Table 5 lists the features of the causal path, including the coefficients of this integrated model and the hypothesis test results. Table 5 demonstrates the entire dataset supports this integrated structure.

**TABLE 5:** Results of hypotheses tests (all samples).

Hypothesis	Route	*T*-Value	Path coefficients
H1a	SYQ → US	7.400	0.237***
H1b	SYQ → PE	13.084	0.392***
H2a	IQ → US	10.469	0.351***
H2b	IQ → PE	12.512	0.377***
H3a	SEQ → US	1.609	0.042
H3b	SEQ → PE	–0.270	–0.007
H4a	PE → US	10.129	0.395***
H4b	US → UI	2.351	0.108*
H5a	SA → IT	12.539	0.375***
H5b	PPT → IT	11.623	0.332***
H5c	FR → IT	11.217	0.331***
H6a	IT → PE	13.441	0.370***
H6b	IT → UI	2.549	0.087*
H7	PE → UI	4.332	0.225***
H8	EE → UI	–0.122	–0.004
H9	SI → UI	5.144	0.168***
H10	FC → UI	2.330	0.088*
H11	HM → UI	4.246	0.143***
H12	PV → UI	6.257	0.211***
H13	HA → UI	3.858	0.136***

*p-value < 0.05; **p-value < 0.01; and ***p-value < 0.001.

In Table 4, the comprehensive structure was examined by WeChat Pay’s Chinese consumer samples in Korea, showing the integrated model is supported. According to the result of Table 4, four paths (H3a, H3b, H6b, and H8; *p*-value > 0.05) are not supported, and the rest of the paths are significant below the 0.05 level. Table 4 normalizes the path coefficients, lists the causal path’s characteristics, and confirms the results of the hypothesis model. Moreover, the comprehensive model was analyzed with Kakao Pay’s Korean consumer samples in Korea (Table 6).

**TABLE 6:** Results of hypotheses tests (Kakao Pay’s Korean consumers sample in Korea).

Hypothesis	Route	*T*-Value	Path coefficients
H1a	SYQ → US	5.007	0.233***
H1b	SYQ → PE	9.275	0.387***
H2a	IQ → US	7.319	0.357***
H2b	IQ → PE	9.704	0.417***
H3a	SEQ → US	1.154	0.045
H3b	SEQ → PE	1.284	0.048
H4a	PE → US	6.989	0.381***
H4b	US → UI	1.171	0.061
H5a	SA → IT	7.888	0.369***
H5b	PPT → IT	7.602	0.332***
H5c	FR → IT	6.677	0.304***
H6a	IT → PE	11.099	0.460***
H6b	IT → UI	11.203	0.509***
H7	PE → UI	0.854	0.050
H8	EE → UI	1.053	0.040
H9	SI → UI	5.520	0.213***
H10	FC → UI	6.747	0.278***
H11	HM → UI	2.568	0.099*
H12	PV → UI	8.161	0.330***
H13	HA → UI	2.566	0.097*

*p-value < 0.05 and ***p-value < 0.001.

Table 6 normalizes the path coefficients, lists the causal path characteristics, and confirms the results of the hypothesis model. The empirical analysis results of Kakao Pay’s Korean consumer samples are shown in Table 6, confirming the existence of the comprehensive model. Taking Kakao Pay’s Korean consumers’ samples as an example, five paths (H3a, H3b, H4b, H7, and H8) are not supported, and the rest of the paths are significant below the 0.05 level.

### Analysis of the Differences in Path Coefficients Between WeChat Pay’s and Kakao Pay’s Korean Groups

The research also studies the mediating effect between WeChat Pay’s Chinese users and Kakao Pay’s Korean users’ groups in Korea. There are two advantages in comparing these two consumer groups. First, WeChat Pay’s Chinese consumers (Chinese tourists in Korea) and Kakao Pay’s Korean consumers (Local Korean in Korea) represent two distinct and distinct demographic features according to income levels, purchasing power, and total accepted qualities of Chinese users. Second, Chinese users play an extremely vital role in m-payments. While Kakao Pay consumers are made up of local Korean users who are more familiar with Kakao Pay than their Chinese tourists’ counterparts, WeChat Pay’s usage model is quite different. Therefore, comparing the comments of Chinese users and local Korean users may lead to a better understanding of usage intention. Based on this manuscript, PE, EE, SI, FC, and ITM are the elements directly determining user willingness, so we studied them further. The different roles of WeChat Pay’s and Kakao Pay’s Korean consumer groups should mitigate the influence of these elements on usage willingness.

The empirical results of hypothesis regulation (H4–H13) are shown in Table 7. First of all, judged by the *p*-values of SI and EE on usage intention, the moderating effects of WeChat Pay and Kakao Pay groups are not significant. Second, the other seven *p*-values show differences in the regulatory effect between the WeChat Pay and Kakao Pay groups. In the WeChat Pay’s Chinese user groups, US (β = 0.351, *p* < 0.01), PE (β = 0.291, *p* < 0.01), HM (β = 0.269, *p* < 0.01), and HA (β = 0.285, *p* < 0.01) positively influence usage intention at a 5% basic level, unlike the Kakao Pay’s Korean groups. In contrast, IT (β = 0.509, *p* < 0.01), FC (β = 0.278, *p* < 0.01), and PV (β = 0.330, *p* < 0.01) positively affected the basic level of usage intention at 1% in Kakao Pay’s Korean consumer group, unlike in the WeChat Pay’s Chinese users’ group.

**TABLE 7:** The difference of path coefficients between WeChat Pay’s and Kakao Pay’s different consumers.

Hypothesis	Route	WeChat Pay		Kakao Pay		Pairwise parameter comparisons	
		β	*P*	β	*P*	*T* value	*p*-Value
H6b	IT → UI	0.057	0.177	0.509	***	7.854	0.000
H4b	US → UI	0.351	***	0.061	0.241	–2.879	0.004
H7	PE → UI	0.291	***	0.050	0.393	–2.704	0.007
H8	EE → UI	0.020	0.593	0.040	0.292	0.564	0.573
H9	SI → UI	0.189	***	0.213	***	1.470	0.142
H10	FC → UI	0.107	0.008*	0.278	***	3.106	0.002
H11	HM → UI	0.269	***	0.099	0.010*	–2.536	0.012
H12	PV → UI	0.079	0.032*	0.330	***	5.193	0.000
H13	HA → UI	0.285	***	0.097	0.010*	–2.951	0.003

*p-value < 0.05 and ***p-value < 0.001.

## Discussion

This study took WeChat Pay and Kakao Pay as the research objects and completely analyzed most elements influencing the sustainable growth of Korean m-payments. Thus far, there has been no theoretical framework comprehensively examining the common influence of various qualities, IT, and technology elements on customer acceptance of m-payment platforms. Additionally, most previous studies (Afshan and Sharif, 2016; Baabdullah et al., 2019) treated customers as an indivisible whole sample group, and few studies examined the regulatory role of different cultural platform features in the trust construction process of the m-payment market. Embracing the concept of integration models will help refine the present literature, since there is no recent research that has been able to combine the successful ITM and D&M models into UTAUT2 to form a combined model to examine the elements affecting the mpayment’s usage intention. After the empirical analysis of the path results proposed in the model, through the multi-group comparison between China and South Korea, we conduct a multi-group test on the cultural effect of the model. In other words, the cultural impact on the path coefficient is tested according to the pairwise parameter comparisons test between the structural loads of two countries.

Hypothesis 14 maintained the influence of IT on usage intention was less for WeChat Pay than for Kakao Pay. The results show initial trust has little positive impact on the intention to use WeChat Pay (β = 0.057, *p* = 0177) than Kakao Pay (β = 0.509, *p* < 0.001). Thus, H14 was supported.

In Hypothesis 15, for Kakao Pay, US on usage intention (β = 0.061, *p* = 0.241) does not significantly affect the intention to use, and in WeChat Pay, satisfaction (β = 0.351, *p* < 0.001) significantly affects the intention to use. Therefore, it is verified that satisfaction in WeChat Pay positively influences usage willingness. Still, for Kakao Pay, US does not affect usage intention. Thus, H15 was supported.

Hypothesis 16 contended that the influence of PE on usage intention would be greater for WeChat Pay users than for the Kakao Pay users. To test H16, the path coefficient between PE and trust was first checked and proved to be statistically significant in the two m-payment systems (shown in Table 7). As stated in H16, the impact of PE on usage intention for WeChat Pay (β = 0.291, *p* < 0.001) was stronger than for Kakao Pay (β = 0.050, *p* = 0.393). This result means the perceived PEs of the technology may affect usage intention. Thus, H16 was supported.

Hypothesis 17 also assumed that there are differences in the relationship between EE and usage intention. Both countries used the same analytics program: effort expectations had a small positive impact on users’ trust for WeChat Pay (β = 0.020, *p* = 0.593) than for Kakao Pay (β = 0.040, *p* = 0.292). As predicted in H17, although the EE of technology’s adoption becomes an important component of trust formation, the relationship between them can be influenced by culture. Thus, we failed to demonstrate the influence of culture on effort expectations and usage intention. Even if the effect of EE on usage intention might vary across cultures, the extent of the difference is too weak to support the expected cultural effect. To some extent, these findings are consistent with previous studies (Bandyopadhyay and Fraccastoro, 2007; Im et al., 2011). Thus, H17 was rejected.

In Hypothesis 18, the influence of SI on use intention is less significant in WeChat Pay than in Kakao Pay. However, as shown in the analytical results in Table 7, the SI on usage intention is significant for WeChat Pay (β = 0.189, *p* < 0.01), whereas it is also significant for Kakao Pay (β = 0.213, *p* < 0.01). That is, for Kakao Pay Korean consumers who are more sensitive to social pressure, SI appears to be an important factor in developing behavioral intentions, whereas, for WeChat Pay Chinese consumers who are more focused on personal goals, the opinions or pressures of others may not be important in their decision-making. Thus, H18 was supported.

In Hypothesis 19, for Kakao Pay, the facilitating condition on the usage intention (β = 0.278, *p* < 0.001) significantly affects the usage intention, and in WeChat Pay, the facilitating condition (β = 0.107, *p* = 0.008) does not significantly affect the usage intention. Therefore, Kakao Pay’s FCs are considered to significantly affect the usage intention, and WeChat Pay’s FCs are verified not to affect the usage intention. Thus, H19 was supported.

In Hypothesis 20, for Kakao Pay, the hedonic motivation on usage intention (β = 0.099, *p* = 0.010) does not significantly affect the intention to use, and in WeChat Pay, hedonic motivation (β = 0.269, *p* < 0.001) significantly affects the intention to use. Therefore, Kakao Pay’s hedonic motivation was considered to affect usage intention significantly, and WeChat Pay’s hedonic motivation was verified to not significantly affect the usage intention. Thus, H20 was supported.

In Hypothesis 21, for Kakao Pay, the PV on usage intention (β = 0.330, *p* < 0.001) significantly affects the intention to use, and in WeChat Pay, the value of the price (β = 0.079, *p* = 0.032) does not significantly affect the intention to use. Therefore, the PV of Kakao Pay is considered to affect the intention to use significantly, and the PV of WeChat Pay is verified to not significantly affect usage intention. Thus, H21 was supported.

In Hypothesis 22, for Kakao Pay, habit on usage intention (β = 0.097, *p* = 0.010) cannot positively affect the usage intention, and in WeChat Pay, habit (β = 0.285, *p* < 0.001) positively affects the usage intention. Therefore, Kakao Pay’s habit is considered to affect usage intent significantly, and WeChat Pay’s habit is not verified to significantly affect usage intention. Thus, H22 was supported.

## Conclusion

Our research conclusions are valuable to researchers and practitioners in the m-payment industry. For the former, this research provides a basis in systematically improving the theoretical model of acceptance, and is a new basis for the theoretical research of new technology acceptance in the future. For practitioners, focusing on the key aspects of the research model is very important for designing, upgrading, and implementing m-payment technologies that can produce high acceptance.

### Theoretical Contribution

Integrating several models and different theories into a comprehensive model, used as an operational framework, it may be helpful to identify potentially important variables between behavior and intentions. We think D&M ISS and ITM are critical elements of UTAUT2, and different researchers have looked for factors that determine usage intention. However, these models are seldom integrated into an integrated model. To solve the shortage of research in related fields, a successful information system model (D&M ISS and ITM) is combined with UTAUT2, and the integration model is used as the conceptual model in this manuscript. The integrated model makes up for the shortcomings of the three separated construers, as well as fully considers the subjective and objective elements of usage intention for two different m-payment apps. Therefore, the contribution is trifold.

First, the above results reveal the integrated model provides a stronger interpretation of usage intention than the ISS model, ITM, or UTAUT2, separately. In other words, we believe that the integrated model offers more demonstrative insights than using a single model view. Consequently, future SEM studies should take a comprehensive viewpoint to test the usage intention of any m-payment platform. This manuscript combined D&M ISS and ITM with UTAUT2 to confirm the influencing factors of the usage intention for m-payments. We found ISS and ITM not only directly affect usage intention, but also through US and PEs alone. The PE’s influence can be the basis and important starting point of future research.

Second, few academics have concentrated on the willingness of potential Chinese users to choose either of the WeChat Pay and Kakao Pay. This is an m-payment research gap which has not been studied at all. This comparison technique increases the effectiveness of several scenarios testing between China and Korea, and also fulfills new specific blanks in m-payment research. We also considered the moderating variables and multi-group analysis of the Chinese and Korean m-payment platforms’ differences to improve the multi-model integration method. The limitation of m-payment knowledge is increased by checking the adjustment variables with the Chinese and Korean payment difference regulators.

Third, attributable to the limited research on the UTAUT2 model for m-payment technology, this study is one attempt to improve UTAUT2 in a multi-model and multi-group integration perspective. In particular, it should be noted that UTAUT2 was originally improved only to examine the use intention of new technology and extended the application of the core variables in the UTAUT2 model to other theoretical fields, such as the cross-cultural background research of consumers, which is composed of many technology platforms and application apps.

### Managerial Implications

Judging by a large amount of m-payment literature, it is clear in different business environments, the factors influencing m-payment adoption intentions vary, so it is necessary to study m-commerce adoption intentions and treat them differently, judged by different countries (Zhang et al., 2012). Under the background of global economic integration, different m-payment providers tend to operate in multiple countries, so it is very important to adopt appropriate strategies to promote m-payment solutions. In general, given the extensive cooperation between WeChat Pay and Kakao Pay, which originally began in early 2017, the two sides have a very broad space for cooperation. By comparing the two m-payment users’ usage intentions, this manuscript aims to focus on vital elements that significantly influence the elements of the two countries’ respective consumers and also provides a necessary theoretical basis and practice for large-scale sustainable cooperation in the m-payment markets of China and Korea.

According to the results of UTAUT2, we offer some practical guidelines for m-payment platform operators and developers. On the one hand, Kakao Pay’s Korean consumers’ IT (H6b), convenience (H10), and PV (H12) are more sensitive, which Chinese mobile operators should focus on, such as establishing transaction privacy protection, business reputation promotion (IT), providing free Wi-Fi (facilitating conditions), and professional Korean after-sales consulting (PV). On the other hand, when Korean m-payment providers enhance China’s consumer usage intention, the focus should be placed on the sensitive factors of Chinese users. For example, Chinese customers are more sensitive to customer satisfaction (H4b), expected performance (H7), hedonic motives (H11), and habits (H13). Therefore, if Korean m-payment providers employ red envelope incentives (US), developing some entertainment functions, such as QR code, radar, and radio to friends (hedonic motivation), and daily special purchase rights (habit) to stimulate the sensitive elements affecting Chinese users, the usage intention of Kakao Pay will be immediately increased. Chinese students in Korea only pay tuition in the form of currency exchange and international remittance. Problems such as money exchange and poor language communication also affect the payment process and efficiency. By stimulating the corresponding sensitive factors affecting use intention, Chinese and Korean m-payment platforms will be able to efficiently improve the usage intention of their respective users and even benefit cross-border m-payments and economic cooperation in APEC.

### Limitations and Future Work

Although this research has theoretical and managerial contributions, it also has other limitations, which are worthy of further study. Future study directions to be discussed are as follows. First, the questionnaire of this manuscript is collected in Korea, and all the answers are in Korea. Future research could be conducted in some more countries to further test the universality of this proposed integrated model. Future research could apply innovative data science and marketing algorithms (Saura, 2021) to evaluate the age, experience, and gender factors of customers into the theoretical model as moderators to survey whether some differences among different consumer samples can be classified according to these features. Therefore, follow-up studies are needed. Second, we focused on only one Korean m-payment provider in Korea. While Kakao Pay is a representative provider of m-payments in Korea, it does not include every area of worldwide m-payments. To enhance the systematic nature of this study, we aim to compare the results from different countries. Last, we combined D&M ISS and ITM with UTAUT2 to determine the elements affecting the m-payment’s usage intention. Future research can use other theories. Research should test the impact of other elements (perceived value, ease of use, and behavior willingness, etc.) on stimulating users’ continued usage intention of m-payment platforms.

## Data Availability Statement

The raw data supporting the conclusions of this article will be made available by the authors, without undue reservation.

## Ethics Statement

Ethical review and approval were not required for the study on human participants in accordance with the local legislation and institutional requirements. Written informed consent from the patients/participants or patients/participants legal guardian/next of kin was not required to participate in this study in accordance with the national legislation and the institutional requirements.

## Author Contributions

XL, Y-TL, and S-CC: conceptualization. XL, KS, AR, and S-CC: methodology and writing—review and editing. XL and S-CC: formal analysis. XL: investigation. XL, AR, Y-TL, and S-CC: writing—original draft preparation. KS and AR: visualization and supervision. All authors have read and agreed to the published version of the manuscript.

## Conflict of Interest

The authors declare that the research was conducted in the absence of any commercial or financial relationships that could be construed as a potential conflict of interest.

## Publisher’s Note

All claims expressed in this article are solely those of the authors and do not necessarily represent those of their affiliated organizations, or those of the publisher, the editors and the reviewers. Any product that may be evaluated in this article, or claim that may be made by its manufacturer, is not guaranteed or endorsed by the publisher.
